# Supplementary figures and images for: Prevalence and correlates of multidimensional child poverty in India during 2015–2021: A multilevel analysis
Source: PLoS One. 2022 Dec 22;17(12):e0279241. doi: 10.1371/journal.pone.0279241 (PMC9779030; doi:10.1371/journal.pone.0279241)

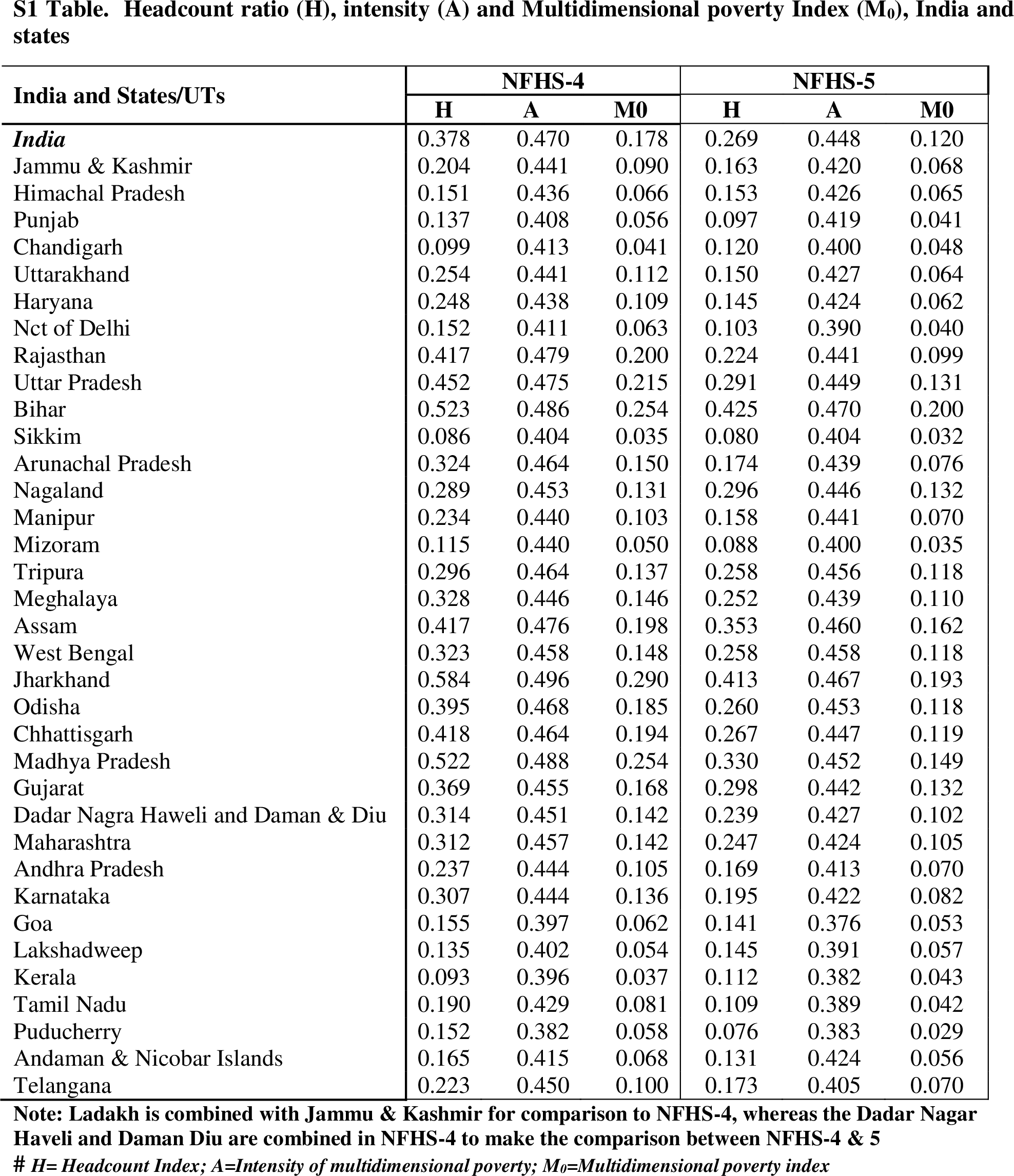

Supplement: S1 Table — (TIF) [file pone.0279241.s001.tif]

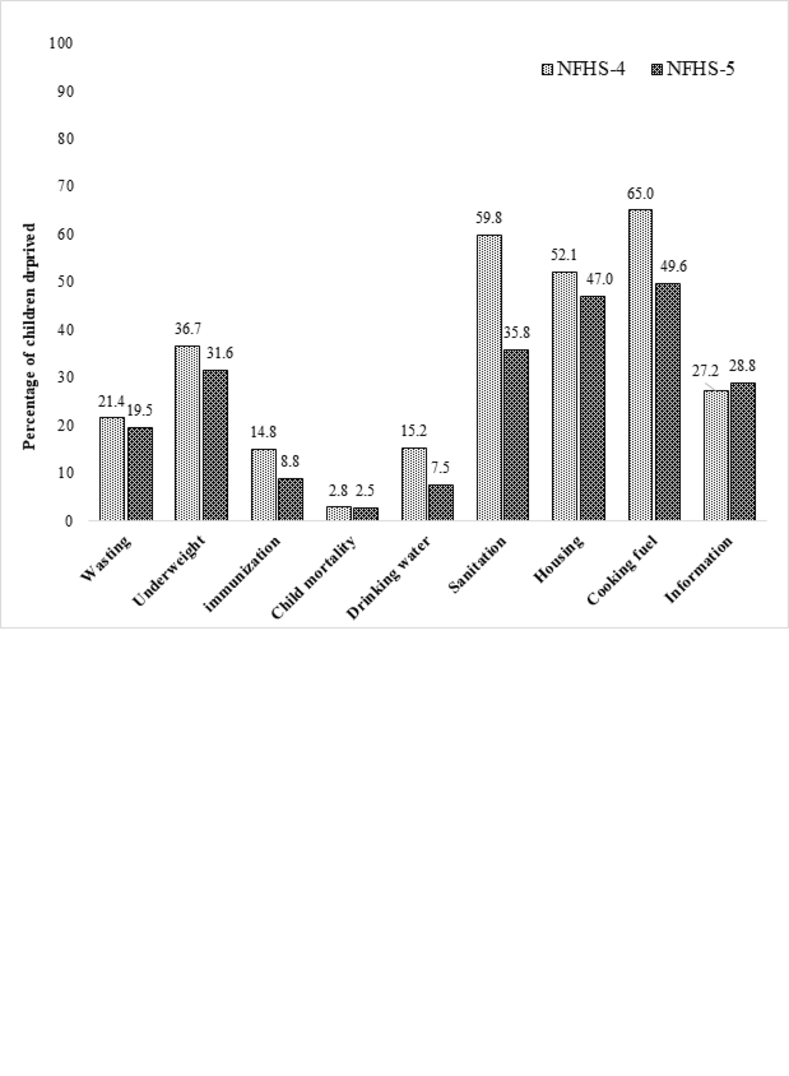

Supplement: S1 Fig — (TIF) [file pone.0279241.s002.tif]
